# Molecular environment in Alzheimer’s disease brain revealed by the hybrid imaging analyses

**DOI:** 10.1002/alz.093362

**Published:** 2025-01-03

**Authors:** Tadayuki Ogawa, Shino Homma‐Takeda, Tomonari Umemura, Takafumi Hirata, Yuko Saito

**Affiliations:** ^1^ Dokkyo Medical University, Mibu, Tochigi Japan; ^2^ National Institute of Quantum Science and Technology, Inage‐ku, Chiba Japan; ^3^ Tokyo University of Pharmacy and Life Sciences, Hachioji, Tokyo Japan; ^4^ University of Tokyo, Bunkyo‐ku, Tokyo Japan; ^5^ Tokyo Metropolitan Geriatric Hospital and Institute of Gerontology, Itabashi‐ku, Tokyo Japan

## Abstract

**Background:**

Although amyloid deposition in brain is one of the hallmark pathological features of Alzheimer’s disease (AD), the upstream events and its molecular environment in AD brain remain largely unknown. Recent advances in analytical methods such as mass spectrometry can provide the cutting‐edge tools to unveil the AD pathogenesis at molecular and atomic level.

**Method:**

In order to gain the comprehensive information about AD pathology at molecular level, postmortem brain sections of AD patients were analyzed by the hybrid molecular imaging methods composed of the conventional histological analyses, matrix assisted laser desorption ionization mass spectrometry imaging (MALDI‐MSI) for small molecules, laser ablation inductively coupled plasma mass spectrometry (LA‐ICP‐MS) imaging for metals, and particle induced X‐ray emission (PIXE) imaging for elements. Principal components analysis (PCA) was also applied to discover the differences between the spectra datasets of AD and healthy control brains. Specific distribution of molecules and elements were visualized on the brain slice. (Ethics Approval: Dokkyo Medical University No.2021‐004, Tokyo Metropolitan Geriatric Hospital and Institute of Gerontology No.R20‐003).

**Result:**

MALDI‐MSI showed abnormal distribution of lipids, such as phospholipids, sphingolipids, and their derivatives in AD brain. LA‐ICP‐MS visualized the distribution of elements, including some metals in the wide‐field brain section. PIXE imaging displayed the specific concentration of atoms nearby the pathological tangles detected by anti‐ubiquitin antibody. PCA proposed the effective classification to distinguish AD from control based on mass spectra.

**Conclusion:**

Our data suggest the molecular and atomic environment in brain that is closely linked to AD pathology. Further analyses should be conducted to elucidate the fundamental mechanism of AD pathogenesis and prevention.

